# Insights into the Mechanisms of Antibody-Affinity Maturation and the Generation of the Memory B-Cell Compartment Using Genetically Altered Mice

**DOI:** 10.1155/1998/42595

**Published:** 1998

**Authors:** Kalpit A. Vora, Jeffrey V. Ravetch, Tim Manser

**Affiliations:** Department of Microbiology and Immunology Kimmel Cancer Institute Thomas Jefferson Medical College Philadelphia PA 19107 USA


*Developmental Immunology*, 1998, Vol. 6, pp. 305–316
Reprints available directly from the publisher
Photocopying permitted by license only

© 1998 OPA (Overseas Publishers Association)
N.V. Published by license under the
Harwood Academic Publishers imprint,
part of the Gordon and Breach Publishing Group
Printed in Malaysia

### *Minireview*

### Insights into the Mechanisms of Antibody-Affinity Maturation and the Generation of the Memory B-Cell Compartment Using Genetically Altered Mice

KALPIT A. VORA, JEFFREY V. RAVETCH and TIM MANSER*

*Department of Microbiology and Immunology, Kimmel Cancer Institute, Thomas Jefferson Medical College, Philadelphia, PA 19107*

*(Received 10 September 1996)*

### INTRODUCTION

The humoral immune system has the ability to acquire memory. Hence, it can recall a previous encounter with an antigen and consequently mount a efficient, specific, and rapid response to a second challenge by that same antigen. A critical aspect of this acquisition of memory is the establishment of a small pool of memory B cells during the latter phases of the primary immune response. In mice and humans, the key characteristics that distinguish antibodies expressed by memory B cells and naive B cells are that memory B cells express somatically hypermutated antibody V regions, and that memory B cells express antibodies that have higher affinities for antigen as compared to antibodies expressed during the primary immune response.

The currently accepted model aimed to explain the recruitment of B cells into the memory compartment suggests that these cells follow a hypermutation and selection pathway. First, genes encoding the variable regions of the Ig molecule are hypermutated (Manser et al., 1985; Rajewsky et al., 1987; Berek and Milstein, 1988). A selection process then results in the preferential expansion of clones that have accumulated mutations that translate into higher affinity for antigen, whereas those V regions that acquire mutations lowering affinity are lost (Eisen, 1966; Siskind and Benaceraff, 1969; Berek et al., 1985; Sablitzky et al., 1985; Liu et al., 1989; Weiss and Rajewsky; 1990). This process may operate in an iterative fashion, that is, the optimal mutation phase is one in which periods of rapid mutation alternate with periods of mutation-free growth (Kepler and Perelson, 1993). Recent versions of the mutation-selection model incorporate the germinal center (GC) microenvironment as sites for recruitment of B-cell clones into the memory pool (Coico et al., 1983; Berek et al., 1991; Jacob et al., 1991a; Linton et al., 1992). GCs are areas of rapid B-cell proliferation arising in primary

*Corresponding author.

lymphoid follicles in response to antigenic stimulation.

### THE GERMINAL CENTER REACTION

Nossal et al. (1964) first demonstrated that the deposition of labeled antigen in lymphoid follicles preceded the formation of GCs. Subsequent experiments demonstrated that injection of mice with preformed immune complexes (IC) accelerated GC formation, the generation of memory B cells, and antibody-affinity maturation (Laissue et al., 1971; Kunkl and Klaus, 1981). Therefore, it has been postulated that the deposition of antigen complexed with antibody (IC) in follicles nucleates the GC reaction. Follicular dendritic cells (FDC) are known to trap IC in the follicles via their complement (CR) and Fc receptors (Tew and Mandel, 1979; Szakal et al., 1989; Yoshida et al., 1993). Depletion of the C3 component of complement with cobra venom factor inhibits GC formation, underscoring the importance of CR in the GC reaction (Pepys, 1976; Klaus and Humphrey, 1977). Recently, a group of cells called "antigen-transporting cells" have been described, which are postulated to transport the IC from the subcapsular sinuses of the lymph nodes and high endothelial venules to the lymphoid follicles (Szakal et al., 1983; Maeda et al., 1995). These cells could be the precursors of FDCs that are thought to play a central role in the GC pathway.

The B-cell oligoclonality of the GC was first suggested by the studies of Kroese and co-workers. Using irradiation chimeras, this group demonstrated that the GC is populated by no more than three B-cell clones (Kroese et al., 1987). A similar conclusion was made by MacLennan and his co-workers studying simultaneous immune responses to two haptens (Liu et al., 1991). In addition, microdissecton of single GCs and PCR amplification of their expressed V genes by Kelsoe's group in mice and Rajewsky's group in humans, unequivocally demonstrated the oligoclonality of GC (Jacob et al., 1991a; Jacob and Kelsoe, 1992; Kuppers et al., 1993).

T cells are the other important constituent of the GC reaction, as SCID mice or irradiated mice reconstituted with B cells alone do not form GC (Von der Heide and Hunt, 1990; Linton et al., 1992). Disruption of cognate B-cell-T-cell interaction by *in vivo* administration of anti-CD40 ligand antibody (Gray et al., 1994) or soluble CD40 ligand (Lane et al., 1993), as well as studies in mice deficient for CD40 (Kawabe et al., 1994) or CD40L (Renshaw et al., 1994; Xu et al., 1994) have shown that this ligand-receptor pair is required for generation of humoral immune responses (primary and secondary) and establishment of the GC reaction. Also, the T cell costimulatory molecule CD28 is necessary for the formation of GCs, as CD28-deficient mice accumulate antigen-reactive cells within the follicles on immunization, but these cells appear to fail to undergo the proliferative expansion necessary to create GC (Ferguson et al., 1996). Recently, it has been demonstrated that T-helper cells in murine GC are clonally related to those found in the PALS, raising the possibility that GC T cells, perhaps like B cells, are antigen-activated emigrants from the PALS (Zheng et al., 1996).

### GERMINAL CENTERS ARE A SITE OF HYPERMUTATION AND AFFINITY MATURATION

Once a GC is established, the B cells populating it undergo extensive proliferation and clonal expansion. Anatomically, GCs can be subdivided into dark and light zones in certain animal species (Liu et al., 1991). The dark zone consists of rapidly dividing blast cells called centroblasts. Concurrent with the establishment of the dark zone around day 7 after antigenic stimulation, somatic hypermutation of V regions expressed by GC B cells is initiated. Studies with FACS-purified GC B cells isolated from mouse spleen (Berek et al., 1991) and human tonsils (Pascual et al., 1994), and microdissected mouse (Jacob et al., 1991b) and human (Kuppers et al., 1993) GC clearly established that GCs are a site for hypermutation. Mutations appear to be introduced in a stepwise manner into the V regions of the dark-zone centroblast, which are in rapid cell cycle but express low levels of surface Ig (Liu et al., 1991). The centroblast numbers do not increase, as half of their progeny appear to exit cell cycle, and reexpress sIg, becoming centrocytes (Hanna, 1964). The centrocytes are smaller and less densely packed than centroblasts, as they are separated by a dense FDC network. Hence, this subanatomical site of the GC is referred to as the light zone (Hardie et al., 1993).

It has been postulated that centrocytes are tested for the ability to bind with increased affinity to the antigen present in IC retained on the FDC network in the light zone (Liu et al., 1991; MacLennan, 1994). Cells that fail to bind die via apoptosis; cells that do bind either reenter the mutation-selection cycle or become memory cells. It has been deduced from the finding of tingible body containing macrophages in the GC that there is a high death rate of cells by apoptosis among centrocytes (Fliendner, 1967). Isolated centrocytes die rapidly by apoptosis on *in vitro* culture unless rescued by surface Ig cross-linking or signals delivered by cognate T-cell help (Liu et al., 1989). Also, it has been shown that resting B cells specific for a particular hapten, if coincubated with FDC-bearing IC containing that hapten, are activated to express higher levels of Ia and costimulatory molecule B7 (Kosco-Vilbois et al., 1993). A similar mechanism could be envisaged for the GC centrocytes. If after mutation the centrocytes express sIg with a higher affinity for antigen, then they could internalize the complexed antigen present on the FDC surface, process it, and present it to antigen-specific GC T-helper cells, and in return receive T-cell help and cell-survival signals, thus avoiding cell death by apoptosis.

### A MODEL SYSTEM TO STUDY THE ROLE OF AFFINITY IN CLONAL DECISIONS TO ENTER THE AFC OR MEMORY B-CELL DIFFERENTIATION PATHWAYS

Clonal selection models for maturation of the B-cell response assume that the degree of B-cell proliferation and differentiation is mirrored by relative sIg affinity for antigen. A threshold affinity is required for a clone to be selected from the naive population to participate in an immune response and be recruited to enter the mutation and selection pathway (Klinman, 1972; Fish et al., 1991). However, the molecular mechanisms that translate relative affinity differences into proliferation advantage or preferred entry into the memory compartment are a subject of speculation. A direct test of the role of affinity in the development of the memory B-cell compartment could be carried out by altering the preimmune antibody repertoire with the introduction of a high-affinity "memory-type" antigen receptor using transgene technology. Earlier work from our laboratory has identified a recombination pathway by which a copy of an antibody $V_H$ transgene recombines with the endogenous IgH locus, forming a transgene-IgH "hybrid" locus (Giusti et al., 1992; Tumas-Brundage et al., 1996). This takes place via a mechanism of interchromosomal, "one-sided" homologous recombination nucleated in a region of mutual homology between one copy of the transgenic array and the endogenous IgH locus. The process is nonreciprocal, and the $V_HDJ_H$ gene DNA in the resulting hybrid locus is always completely transgene-derived. The recombination occurs at a very low frequency in the B-cell compartment. Consequently, only a small fraction of B cells express the hybrid locus in transgenic animals, whereas B cells in which this recombination has not taken place express only endogenous V genes.

In these studies, we used a model immune response to arsonate in A/J mice. During this immune response, a family of antibodies encoded by a single combination of $V_H$ (VhIdCR), D (DFL16.1), $J_H$(Jh2), Vk(VkIdCR/Vk10), and Jk(Jk1) gene segments become predominant (Manser et al., 1987). This combination of gene segments encodes "canonical" antiarsonate antibodies, the prototype of which is expressed by the hybridoma 36-65. During an anti-Ars immune response, canonical antibody expressing B cells undergo hypermutation and antigen-affinity-based selection, resulting in the expression of somatically mutated antibodies having a 5- to 20-fold increased affinity for arsonate (Manser et al., 1987; Sharon et al., 1989).

We generated transgenic mice with a somatically mutated "memory-type" $V_H$ gene called 36-71 that, in combination with a canonical $\kappa$ chain, encodes a canonical antibody with 100× higher affinity than the unmutated canonical antibody 36-65 (Sharon, 1990; Vora and Manser, 1995). The $V_H$36-71 transgenic mice have a very low frequency of cells in their naive B-cell compartment expressing transgene-IgH hybrid loci, and this frequency is similar to that found for B cells expressing endogenous canonical $V_H$ gene segments (Vora and Manser, 1995). This establishes the scenario for studying the effects of surface Ig affinity on a B-cell clone's participation in the immune response. A number of possibilities could be envisaged for the participation of the high affinity (36-71 transgene-expressing) antiarsonate B-cell clones in the response (Figure 1):

1. Transgene-expressing clones might be preferentially stimulated early during the primary response. The efficient capture of soluble antigen due to high surface Ig affinity could focus T-cell help, resulting in terminal differentiation into AFC, perhaps precluding entry into the B-cell memory pool.
2. The high-affinity transgenic clone might have the requisite affinity to be directly recruited to the memory compartment, bypassing the GC reaction or only transiently passing through the GC. The predictions under these conditions would be exclusive participation of the transgene-expressing clone in the secondary immune response, without accumulating any new mutations in its V-gene segments.
3. The transgene-expressing high-affinity B-cell clones could seed the GC reaction very early, resulting in the expression of affinity-matured antibodies early in the primary immune response.
4. The effect of expression of a high-affinity antigen receptor may be purely quantitative, thus allowing dominant participation of the transgene-expressing clones in both the primary AFC and GC/B memory pathways.

Besides the potential *cis* effects of the transgene-encoded antibody on the outcome of the immune response among transgene-expressing clones, a *trans* effect of a transgene-encoded antibody on the participation of clones expressing endogenous antibody genes could also be exerted. For example, the participation of high-affinity transgene clones could exclude or reduce the participation of lower-affinity endogenous $V_H$ expressing clones via cellular competition. In addition, the serum antibody produced by the high-affinity clones could indirectly alter the participation of lower-affinity clones.

The results we obtained supported model number four, and clearly demonstrated a lack of either *cis* or *trans* effects of $V_H$ 36-71 transgene expression on (a) the kinetics of serum antibody production; (b) the extent and type of isotype switching; and (c) the degree of V-gene mutation (Vora and Manser, 1995). That is, all these processes were indistinguishable in transgene-expressing and endogenous V-gene-expressing canonical antiarsonate clones. The separation of the mutational process (occurring exclusively in the GC dark-zone centroblasts) from the affinity-based selection process (occurring in the GC light zone), as proposed by MacLennan, could account for the lack of a *cis* influence of surface Ig affinity on the hypermutation process (Liu et al., 1991). The reasons for the apparent lack of *cis* influences on class switching and on the kinetics of serum antibody production are currently under investigation.

A major fraction of primary antiarsonate serum antibodies induced in $V_H$36-71 transgenic mice appeared to be transgene-encoded, excluding model number two. Nonetheless, antiarsonate antibodies that were not transgene-encoded were also abundant, demonstrating the the participation of high-affinity transgene clones in the AFC response does not exclude endogenous clones from participating in this response. Similarly, approximately equal numbers of hybridomas expressing transgene-encoded and endogenous canonical antibodies were isolated from secondary arsonate responses, excluding model number one, and indicating that the participation of transgene-expressing clones in the GC/memory pathway does not alter participation of endogenous clones

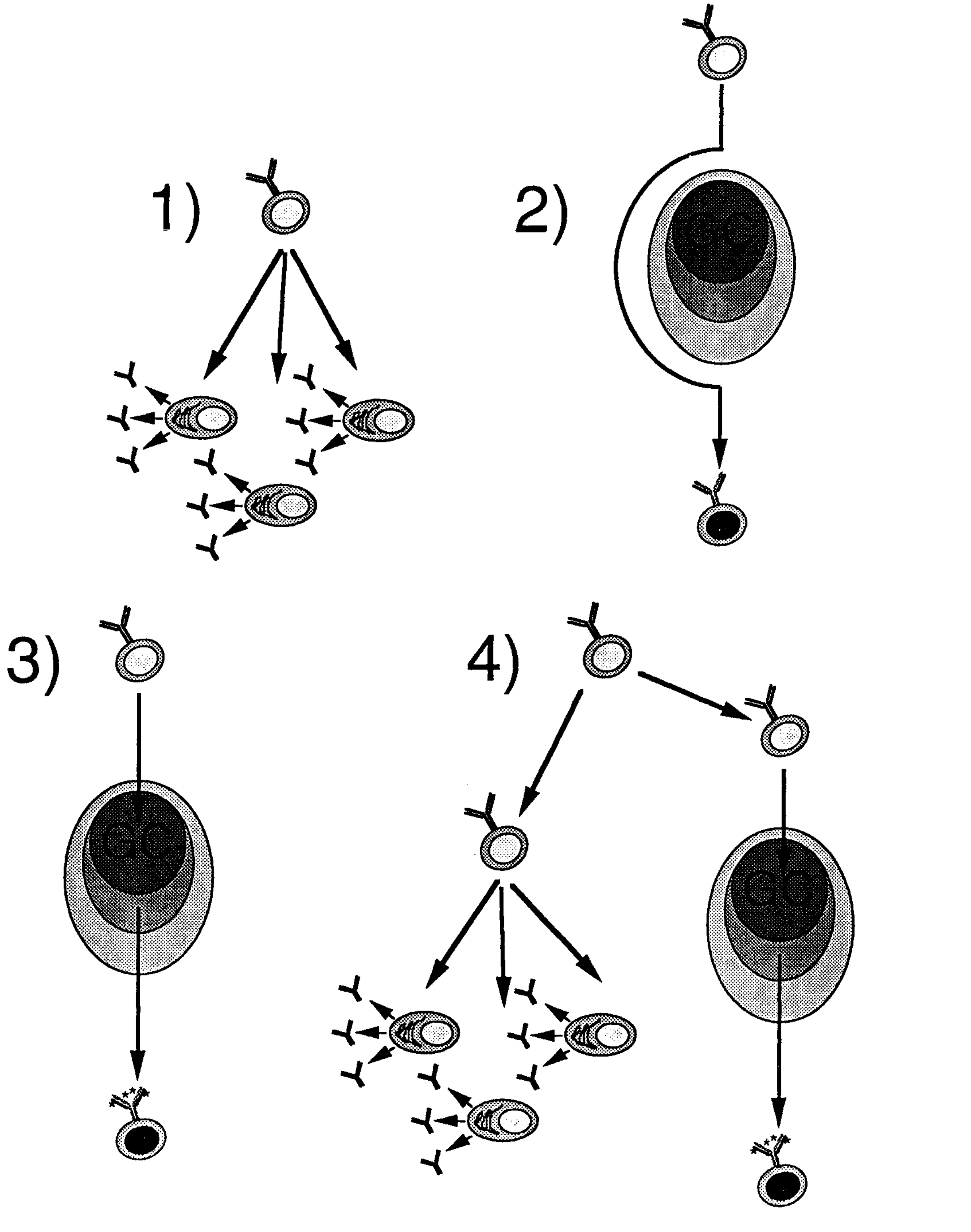

FIGURE 1 Potential fates of a preimmune B cells expressing a somatically mutated, high-affinity variable region. Germinal centers are indicated by "GC"; V-region somatic mutations are indicated by asterisks. "Naive" B cells are shown containing light-gray nuclei. Memory B cells are shown containing black nuclei. AFC are depicted as ellipsoid cells. Four possible differentiative pathways are shown: (1) proliferation and direct differentiation into AFC; (2) direct entry into the memory compartment, by passing or transiently passing through germinal centers; (3) exclusive and preferential entry into germinal centers; and (4) participation in both GC/B memory and AFC pathways.

in this pathway. The most intriguing observation we made from the studies on $V_H$36-71 transgenic mice was that both transgene-encoded and endogenous V-gene-encoded antibodies expressed by hybridomas isolated after secondary immunization had a 2-to 30-fold higher affinity for arsonate than their unmutated precursors. This was a particularly striking result given the fact that at the onset of the immune response, transgene-expressing B-cell clones express antibodies with an affinity for arsonate 100× higher than endogenous antiarsonate B-cell clones.

These observations might be reconciled by the assumption that affinity maturation in transgene-expressing and endogenous V-region-expressing clones always takes place in different GCs (Figure 2). The pauciclonality of the GC reaction during the mutation-selection period would make it extremely unlikely that distinct antiarsonate B-cell clones would be present in the same GC at the same time (Kroese et al., 1987; Liu et al., 1991; Jacob and Kelsoe, 1992). Physical isolation of multiple clones expressing antibodies specific for same epitope but of different affinities would allow "clone-autonomous" maturation and affinity-based memory B-cell selection (Figure 2).

As described before, it has been postulated that the deposition of IC on the surface of FDC nucleates the GC reaction (Nossal, 1994). In arsonate-immunized $V_H$36-71 transgenic mice, this antibody should be of polyclonal origin, that is, a mixture of low (endogenous) and high (transgenic) affinities. If "testing" of the affinity of antibodies expressed by GC B cells relative to the polyclonal Ig present in FDC-bound ICs is involved in affinity maturation, we should have observed a "normalization" of the affinity of antibodies produced by transgene-expressing and endogenous V-gene-expressing clones. This result was not obtained.

These observations do not, at face value, support the current idea that affinity-based antigen selection takes place via interaction of B cells with FDC-bound ICs. However, it is possible that during the initial selection-proliferation phase of the GC reaction (before the onset of hypermutation), recruited B-cell clones secrete antibody that binds locally to a limited number of available epitopes on FDC-bound ICs that were generated during the AFC phase of the response. After hypermutation, competition for binding to this limited number of sites between the previously secreted antibody and newly emerging mutant antigen receptors could allow selection of mutants with increased affinity in a "clone-autonomous" fashion. A more extensive analysis of interaction between B cells and FDCs during the GC reaction, and determination of whether affinity-based selection takes place as a consequence of this interaction will be required to gain further insight into the mechanism of clone-autonomous affinity maturation and memory B-cell generation.

### A MODEL SYSTEM TO STUDY THE ROLE OF FDC-BOUND IMMUNE COMPLEXES IN THE GC REACTION, AFFINITY MATURATION, AND THE GENERATION OF B-CELL MEMORY

As described earlier, a significant amount of emphasis has been placed on the role of ICs in the initiation of the GC and in the affinity-based selection of V-region somatic mutants. According to the current models, formation and deposition of ICs on the surface of FDCs in B-cell follicles creates the follicular antigen-retaining reticulum (FARR), into which activated memory precursors migrate and undergo extensive proliferation and somatic mutation. B cells are postulated to be selected to become secondary AFC and long-lived memory B cells by competing for native antigen on the FARR (MacLennan, 1994). Long-term retention of antigen on the FARR has also been suggested to play a role in maintaining the memory B-cell population (Nossal, 1964; Tew et al., 1990; Maclennan, 1994; Lindhout and deGroot, 1995). FcRs and CRs present on the FDC are probably involved in the trapping of IC to form the FARR. Evidence in support of the role of CRs in this trapping mechanism and in the regulation of the B-cell response has been obtained. Depleting complement by injection of cobra venom factor inhibited both deposition of ICs on the FARR and B-cell

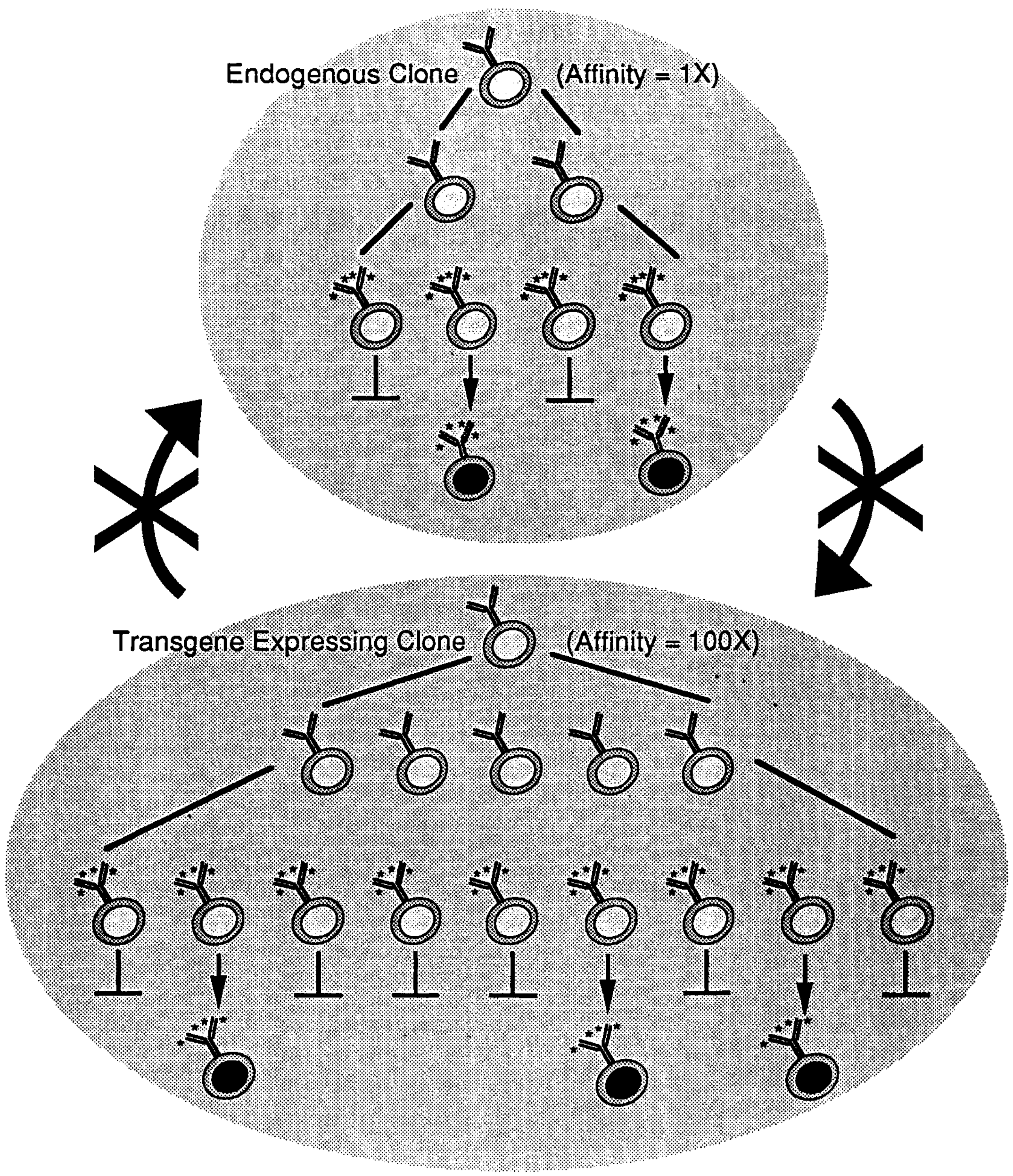

FIGURE 2 Clone autonomous maturation of the B-cell immune response. Two clones are depicted undergoing V-region hypermutation, clonal expansion, and selection in two distinct microenvironments (GCs?). The endogenous clone starts the process with "1×" affinity for arsonate, and the $V_H$36-71 transgene-expressing clone starts the process with an affinity for arsonate 100-fold higher ("100×"). Memory-cell precursors are indicated with light nuclei and memory cells are indicated with black nuclei. The arrows interrupted by "×s" are meant to indicate that the two microenvironments do not "communicate" with one another regarding affinity for antigen. This "communication" might take place either via soluble antibody or cellular migration. The blocked lines indicate either death or lack of further proliferation by a mutant subclone. We have obtained data suggesting that the high-affinity transgene-expressing clone gives rise to more progeny that have reduced affinity for antigen than does the low, affinity-endogenous clone, and this is depicted here. Nevertheless, both clones appear to contribute approximately equally to the memory B-cell compartment, and this is also indicated.

memory development (Pepys 1976; Klaus and Humphrey, 1977). Blocking complement receptors using mAbs had dramatic effects on B-cell activation *in vitro* and resulted in suppression of immune response *in vivo* (Klaus and Humphrey, 1986; Heyman et al., 1990; Thyphronitis et al., 1991). Recently, it has been shown that CR1 and CR2 knockout mice have a severe defect in their humoral response (Ahearn et al., 1996; Molina et al., 1996).

In contrast, the role of FcR in FDC antigen trapping and regulation of the B-cell response has received less attention. These receptors link humoral (antibodies) and cellular immunity (effector cells), and are present on most effector cells of the immune system (Ravetch and Kinet, 1991). The FcR $\gamma$ chain is required for surface expression of and signal transduction via all murine Fc$\gamma$Rs except Fc$\gamma$RIIB (Ravetch and Kinet, 1991), the only Fc$\gamma$R expressed by B cells. Therefore, we chose to examine the B-cell immune response in mice with a targeted inactivation of the FcR $\gamma$-chain gene (Takai et al., 1994). We felt that the FcR $\gamma^{-/-}$ mutation would be particularly informative to study, as the expression of Fc$\gamma$RIIB on B cells is not affected by this mutation, and, hence, any alteration of the B-cell response in such mice could be reasonably attributed to "trans" effects mediated by non-B cells.

The first question we addressed using the $\gamma^{-/-}$ mice was whether IC-trapping capacity of FDCs in the follicles had been perturbed. We passively generated ICs by injecting rabbit anti-horse radish peroxidase (HRP) serum, followed by injecting HRP a day later (Vora et al., 1997). Immunohistological examination of the spleens of $\gamma^{-/-}$ mice and control heterozygous litter mates revealed substantial increases in both the frequency and the intensity of FARR staining in the $-/-$ mice. We estimated that the frequency of the visible HRP FARR per spleen was at least tenfold increased in the $\gamma^{-/-}$ mice. Also, on average 7.5-fold more HRP was present in the numerous individual FARR of the $\gamma^{-/-}$ mice, as compared to the infrequent FARR present in control mice.

The enhanced deposition of HRP colocalized with regions that also stained with the mAb FDC-M1 (Tew et al., 1990), specific for FDC, strongly indicating that this enhanced deposition was on FDC. Since cell-surface expression of the Fc receptors Fc$\gamma$RI and Fc$\gamma$RIII are ablated in $\gamma^{-/-}$ mice, the localization of ICs on the FDC could be mediated either by CRs, by Fc$\gamma$RIIB, or both types of receptor. Treatment of $\gamma^{-/-}$ mice with cobra venom factor prior to passive generation of ICs *in vivo* led to IC deposition on the FARR being substantially reduced but not abolished. These findings are in keeping with previous reports, indicating that human and mouse FDCs express high levels of CR (Reynes et al., 1985; Schriever et al., 1989; Yoshida et al., 1993).

The mechanism responsible for enhanced IC deposition on the FARR in mice lacking the FcR $\gamma$ chain is presently unclear. The most likely explanation is inefficient clearance of ICs from the circulation due to absence of Fc$\gamma$RI and Fc$\gamma$RIII on phagocytic cells of macrophage/monocytic lineage. This would effectively increase the amount of ICs reaching FARR. Previous studies have implicated marginal zone B cells in active transport of ICs to the FARR by CRs (Brown et al., 1973; Gray et al., 1984; Humphrey et al., 1984; Heinen et al., 1986; Kroese et al., 1986). Since CRs are unaffected in the $\gamma^{-/-}$ mice, the increased availability of IC with fixed complement may dramatically augment the transport of IC to follicles. This line of reasoning ascribes a possible role for FcRs in IC clearance and tissue distribution in addition to their well-documented role in triggering effector responses.

The dramatic increase of IC deposition on FDC in follicles in the $\gamma^{-/-}$ mice makes them an ideal model system to study the influence of antigen concentration and epitope density in the FARR on maturation of the B-cell response and generation and maintenance of B-cell memory. If the current models regarding the role of ICs in initiation of the GC reaction, affinity maturation and maintenance of the memory B-cell compartment are correct, the following predictions should be borne out:

1. The elevated concentration of antigen present on the FARR of $-/-$ mice should lower the stringency of affinity-based selection among B cells expressing somatically mutated antibodies, due to less competition for antigen on or released from the FARR.

2. Quantitative differences in frequency and life span of memory B cells should be observed between $\gamma^{-/-}$ and control mice.
3. The somatic hypermutation process may be altered in $\gamma^{-/-}$ mice as this process could be affected by the increased deposition of antigen on the FARR.

Since the $\gamma^{-/-}$ mice were constructed on a mixed C57BL/6 background, we studied the well-characterized model immune response to the hapten (4-hydroxy-3-nitrophenyl) acetyl (NP) (Jacob et al., 1991b) to test these predictions. We found no evidence for overt alteration of serum antibody production, isotype switching, somatic hypermutation, and affinity maturation in the anti-NP response of $\gamma^{-/-}$ mice. We did observe a slightly accelerated primary GC reaction peaking at day 7 in $\gamma^{-/-}$ mice as compared to days 10-13 in control mice. Previous experiments in which preformed ICs were used as immunogens in mice revealed accelerated GC formation (Laissue et al., 1971; Kunkl and Klaus, 1981). Taken together, these observations suggest that IC formation and deposition can influence the initiation of the GC response.

These results raise questions regarding hypotheses that propose a direct role for ICs and FDCs (that make up the FARR) in the ongoing regulation and magnitude of maturation of the primary B-cell response. Our results support a model in which the initiation of the primary GC reaction, but not subsequent B-cell affinity-based selection and differentiation events, are influenced by B-cell epitope density on the FARR. Mechanisms of affinity-based selection-mediated by the FARR that do not involve direct cellular competition might still be envisioned, however. For example, selection of higher-affinity B-cell variants on the basis of time of interaction with the FDC surface, or selection due to increased surface Ig cross-linking mediated by FDC-bound antigen, as somatically mutated antibody replaces unmutated antibody on the surface of individual B cells.

Alternatively, T-cell help available in the GC could be limiting, allowing stringent selection of higher-affinity antibody variants based on B-cell competition for interaction with GC $T_H$ cells. In this regard, it has been proposed that high-affinity B cells that are generated in the GC can capture FDC-bound antigen, process it, and present it to GC $T_H$ cells, thus obtaining cognate proliferative and survival signals necessary for entry into the memory pool (MacLennan, 1994).

In total, data supporting the role of FARR in the regulation of the primary B-cell response is currently anecdotal and controversial. In fact, some studies suggest that the FARR is not required for GC formation (Kroese et al., 1986). Recently, the analysis of lymphotoxin-$\alpha$-deficient mice has shown that somatic mutation and affinity maturation can take place in the absence of FARR (Matsumato et al., 1996). In contrast, there is a large body of evidence supporting the role of FARR in the induction and maintenance of memory responses (Tew et al., 1980; Gray and Skarvall, 1988; Szakal et al., 1992; Terashima et al., 1992; Bachmann et al., 1994). In light of these latter results, it was surprising that $\gamma^{-/-}$ and control littermates mice did not show any significant differences in serum antibody levels after secondary immunization. Nevertheless, we are currently investigating whether the dramatically increased levels of antigen in the FARR of $\gamma^{-/-}$ mice result in the alteration of long-term memory and serum antibody production.

### CONCLUSIONS

Our studies using $V_H$36-71 transgenic mice have demonstrated that antibody-affinity maturation and the generation of the memory B-cell compartment take place in a "clone-autonomous" fashion. This may result, in part, from the physical isolation of individual antigen-responsive clones in different GCs and the lack of "communication" between these GCs during the primary immune response. Despite the great deal of significance that has been placed on the role of FDC-bound ICs in antigen-driven B-cell development and affinity maturation, our studies using FcR $\gamma^{-/-}$ mice demonstrated that at least a tenfold increase in IC density on the FARR does not result in perceptable alterations in the maturation of the B-cell response and the development of B-cell memory. Currently popular models that seek to explain the maturation of the B-cell immune response should be amended given these results.

### *Acknowledgments*

We would like to thank all the members of the Manser laboratory for their many indirect contributions to this work. This work was supported by grants from the NIH to T.M. and J.V.R. K.A.V. was supported by NIH-training grant 5-T32-CA09678.

### *References*

Ahearn J.M., Fischer M.B., Croix D., Gorrg S., Ma M., Xia J., Zhou X., Howard R.G., Rothstein T.L., and Carroll M.C. (1996). Disruption of the CR2 locus results in a reduction in B1a cells and in an impaired B cell response to T-dependent antigen. Immunity **4**:251-262.

Bachmann M.F., Kundig T.M., Hengartner H., and Zinkernagel R.M. (1994). Regulation of Ig antibody titers by the amount persisting of immune-complexed antigen. Eur. J. Immunol. **24**:2567-2670.

Berek C., Berger A., and Apel M. (1991). Maturation of the immune response in the germinal centers. Cell **67**:1121-1129.

Berek C., Griffiths G.M., and Milstein C. (1985). Molecular events during maturation of the immune response to oxazalone. Nature **316**:412-418.

Berek C., and Milstein C. (1988). The dynamic nature of the antibody repertoire. Immunol. Rev. **105**:5-26.

Brown J.C., Harris G., Papamichail M., Sljivic V.S., and Hollborow E.J. (1973). The localization of aggregated human $\gamma$-globulin in the spleens of normal mice. Immunology **25**:955-968.

Coico R.F., Bhogal B.S., and Thoerbecke G.J. (1983). Relationship of germinal centers in lymphoid tissue to immunological memory. VI. Transfer of B-cell memory with lymph node cells fractionated according to their receptors for peanut agglutinin. J. Immunol. **131**:2254-2257.

Eisen H.N. (1966). The immune response to a single antigenic determinant. Harvey Lectures **60**:1-33.

Ferguson S.E., Han S., Kelsoe G., and Thompson C.B. (1996). CD28 is required for germinal center formation. J. Immunol. **156**:4576-4581.

Fish S., Fleming M., Sharon J., and Manser T. (1991). Different epitope structures select distinct mutant forms of an antibody variable region for expression during the immune response. J. Exp. Med. **173**:665-672.

Fleindner T.M. (1967). On the origin of tingible bodies in germinal centers in immune responses. In Germinal Centers in Immune Responses, Cother, H., ed. (Berlin: Springer-Verlag), pp. 218-224.

Giusti A.M., Coffee R., and Manser T. (1992). Somatic recombination of heavy chain variable region transgenes with the endogenous immunoglobulin heavy chain locus in mice. Proc. Natl. Acad. Sci. USA **89**:10321-10325.

Gray D., Dullfore P., and Jainandensing S. (1994). Memory B cell development but not germinal center formation is impaired by in vivo blockade of CD40-CD40 ligand interaction. J. Exp. Med. **180**:141-155.

Gray D., McConnell I., Kumoraratne D.S., Maclennan I.C.M., Humphrey J.H., and Bazin H. (1984). Marginal zone B cells express CR1 and CR2 receptors. Eur. J. Immunol. **14**:47-52.

Gray D., and Skarvall H. (1988). B-cell memory is short lived in the absence of antigen. Nature **336**:70-73.

Hanna M.G. (1964). An autoradiographic study of the germinal center in spleen while pulp during early intervals of the immune response. Lab. Invest. **13**:95-104.

Hardie D.L., Johnson G.D., Khan M., and MacLennan I.C.M. (1993). Quantitative analysis of molecules which distinguish functional compartments in germinal centers. Eur. J. Immunol. **23**:997-1004.

Heinen E., Braun M., Coulie P.G., Van Snick J., Moeremans M., Cormann N., Kinet-Denoel C., and Simar L.J. (1986). Transfer of immune complexes from lymphocytes to follicular dendritic cells. Eur. J. Immunol. **16**:167-172.

Heyman B., Wiersma E.F., and Kinoshita T. (1990). In vivo inhibition of the antibody response by a complement receptor specific monoclonal antibody. J. Exp. Med. **172**:665-668.

Humphrey J.H., Grennan D., and Sundaram V. (1984). The origin of follicular dendritic cells in the mouse and the mechanism of trapping of immune complexes on them. Eur. J. Immunol. **14**:859-864.

Jacob J., Kassir R., and Kelsoe G. (1991b). In situ studies of the primary immune response to (4-hydroxy-3-nitrophenyl)acetyl. I. The architecture and dynamics of responding cell populations. J. Exp. Med. **173**:1165-1175.

Jacob J., and Kelsoe G. (1992). In situ studies of the primary immune response to (4-hydroxy-3-nitrophenyl)acetyl. II. A common clonal origin for periarteriolar lymphoid sheath associated foci and germinal centers. J. Exp. Med. **176**:679-688.

Jacob J., Kelsoe G., Rajewsky K., and Weiss U. (1991a). Intraclonal generation of antibody mutants in GC. Nature **354**:389-392.

Kawabe T., Naka T., Yoshida K., Tanaka T., Fujiwora H., Suematsu S., Yoshida N., Kishimoto T., and Kikutani H. (1994). The immune response in CD40 deficient mice: Impaired immunoglobulin class switching and germinal center formation. Immunity **1**:167-178.

Kepler T.B., and Pelerson A.S. (1993). Cyclic re-entry of germinal center B cells and the efficiency of affinity maturation. Immunol. Today **14**:412-415.

Klaus G.G.B., and Humphrey J.H. (1977). The generation of memory cells. I. The role of $C_3$ in the generation of B-memory cells. Immunology **33**:31-40.

Klaus G.G.B., and Humphrey J.H. (1986). A re-evaluation of the role of C3 in B-cell activation. Immunol. Today **7**:163.

Klinman N.R. (1972). The mechanism of antigenic stimulation of primary and secondary clonal precursor cells. J. Exp. Med. **136**:241-260.

Kosco-Vilbois M.H., Gray D., Scheidegger D., and Julius M. (1993). Follicular dendritic cells help resting B cells to become effective antigen-presenting cells: Induction of B7/BB1 and upregulation of major histocompatibility complex class II molecules. J. Exp. Med. **178**:2055-2066.

Kroese F.G.M., Wubenna A.S., and Nieuwenhius P. (1986). Germinal center formation and follicular antigen trapping in the spleen of lethally x-irradiated and reconstituted rats. Immunology **57**:99-104.

Kroese F.G.M., Wubenna A.S., Seijen H.G., and Nieuwenhius P. (1987). Germinal centers develop oligoclonally. Eur. J. Immunol. **17**:1069-1072.

Kunkl A., and Klaus G.G.B. (1981). The generation of memory cells. IV. Immunization with antigen-antibody complexes accelerates the development of B-memory cells, the formation of germinal centres and the maturation of antibody affinity in the secondary response. Immunology **43**:371-378.

Kuppers R., Zhao-Hohn M., Hansmann M.-L., and Rajewsky K. (1993). Tracing B cell development in human germinal center by molecular analysis of single cells picked from histological sections. EMBO J. **12**:4955-4967.

Laissue J., Cottier H., Hess M., and Stoner R.D. (1971). Early and enhanced germinal center formation and antibody responses in mice after primary stimulation with antigen isologous antibody complexes as compared with antigen alone. J. Immunol. **107**:822-831.

Lane P., Brocker T., Hybele S., Padovan E., Lanzavecchia A., and McConnel F. (1993). Soluble CD40L can replace the normal T cell derived CD40 ligand signal to B cells in T-cell-dependent activation. J. Exp. Med. **177**:1209-1213.

Lindhout E., and deGroot C. (1995). Follicular dendritic cells and apoptosis: Life and death in germinal centers. Histochem. J. **27**:167-183.

Linton P.-J., Lo D., Lai L., Thorbecke G.J., and Klinman N.R. (1992). Among naive precursor cell populations, only progenitors of memory B cells originate germinal centers. Eur. J. Immunol. **22**:1293-1297.

Liu Y.J., Joshua D.E., Williams G.T., Smith C.A., Gordon J., and MacLennan I.C.M. (1989). Mechanism of antigen-driven selection in germinal centers. Nature **342**:929-931.

Liu Y.J., Zhang J., Lane P.J.L., Chan E.Y.T., and MacLennan I.C.M. (1991). Sites of specific B cells activation in primary and secondary responses to T-cell dependent and T-independent antigens. Eur. J. Immunol. **21**:2951-2962.

MacLennan I.C.M. (1994). Germinal centers. Ann. Rev. Immunol. **12**:117-139.

Maeda K., Ksoco-Vilbois M.H., Burton G.F., Szakal A.K., and Tew J.G. (1995). Expression of the intercellular adhesion molecule-1 on high endothelial venules and on non-lymphoid antigen handling cells: Interdigitating cells, antigen transporting cells and follicular dendritic cells. Cell Tiss. Res. **279**:47-54.

Manser T., Wysocki L.J., Gridely T., Near R.I., and Gefter M.L. (1985). The molecular evolution of the immune response. Immunol. Today **6**:94-101.

Manser T., Wysocki L.J., Margolies M.N., and Gefter M.L. (1987). Evolution of antibody variable region structure during the immune response. Immunol. Rev. **96**:141-162.

Matsumato M., Low S.F., Carruthers C.J.L., Min J., Mariathasan S., Huang G., Plas D.R., Martin S.M., Geha R.F., Nahm M.H., and Chaplin D.D. (1996). Affinity maturation without germinal centers in Lymphotoxin-$\alpha$ deficient mice. Nature **382**:462-466.

Molina H., Holers M.V., Li B., Fang F.-Y., Mariathason S., Goellner J., Strauss-Schoenberger J., Karr R., and Chaplin D.D. (1996). Markedly impaired humoral immune response in mice deficient in complement receptors 1 and 2. Proc. Natl. Acad. Sci. USA **93**:3357-3361.

Nossal G.J.V. (1994). Differentiation of the secondary B-lymphocyte repertoire: The germinal center reaction. Immunol J. Rev. **137**:172-183.

Nossal G.J.V., Ada G.L., and Austin C. (1964). Antigens in immunity. IV. Cellular localization of $^{125}$I and $^{131}$I-labeled flagella in lymph nodes. Aust. J. Exp. Biol. Med. Sci. **42**:311-330.

Pascual V., Liu Y.-J., Magalski A., de Bouteiller O., Banchereau J., and Capra J.D. (1994). Analysis of somatic mutation in B cell subsets of human tonsil correlates with phenotypic differentiation from naive to the memory B cell compartment. J. Exp. Med. **180**:329-339.

Pepys M.B. (1976). Role of complement in the induction of immunological responses. Transplant. Rev. **32**:93-120.

Rajewsky K., Froster E.I., and Cumano A. (1987). Evolutionary and somatic selection of the antibody repertoire in the mouse. Science **238**:1088-1094.

Ravetch J.V., and Kinet J.P. (1991). Fc receptors. Ann. Rev. Immunol. **9**:457-492.

Renshaw B.R., Fanshlow W.C., Armitage R.J., Campbells K.A., Liggitt D., Wright B., Davison B.L., and Maliszewski C.R. (1994). Humoral responses in CD40 ligand-deficient mice. J. Exp. Med. **180**:1889-1900.

Reynes M., Aubert J.P., Cohen J.H.M., Audouin J., Tricollet V., Diebold J., and Kazatchkine M.D. (1985). Human follicular dendritic cells express CR1, CR2 and CR3 complement receptor antigens. J. Immunol. **135**:2687-2694.

Sablitzky F., Wildner G., and Rajewsky K. (1985). Somatic mutation and clonal expansion of B-cells in an antigen driven immune response. EMBO J. **4**:345-350.

Schriever F., Freedman A.S., Freeman G., Messner G., Lee G., Daley J., and Nadler L.M. (1989). Isolated human follicular dendritic cells display a unique antigenic phenotype. J. Exp. Med. **169**:2043-2058.

Sharon J. (1990). Structural correlates of high antibody affinity: Three engineered amino acid substitutions can increase the affinity of an anti-p-azophenylarsonate antibody 200-fold. Proc. Natl Acad. Sci. USA **87**:4814-4817.

Sharon J., Gefter M.L., Wysocki L.J., and Margolies M.N. (1989). Recurrent somatic mutations in mouse antibodies to p-azophenylarsonate increase affinity for hapten. J. Immunol. **142**:596-601.

Siskind G.W., and Benaceraff B. (1969). Cell selection by antigen in the immune response. Adv. Immunol. **10**:1-50.

Szakal A.K., Holmes K.L., and Tew J.G. (1983). Transport of immune complexes from the subcellular sinus to lymph node follicles on the surface of non-phagocytic cells, including cells with dendritic morphology. J. Immunol. **131**:1714-1727.

Szakal A.K., Kapasi Z.F., Masuda A., and Tew J.G. (1992). Follicular dendritic cells in the alternative antigen transport pathway: Microenvironment, cellular events, age, and retrovirus related alteration. Sem. Immunol. **4**:257-266.

Szakal A.K., Kosco M.H., and Tew J.G. (1989). Microanatomy of lymphoid tissue during humoral immune responses: Structure and function relationships. Ann. Rev. Immunol. **7**:91-109.

Takai T., Li M., Sylvestre D., Clynes R., and Ravetch J.V. (1994). FcR$\gamma$ chain deletion results in pleiotrophic effector cell defects. Cell **76**:519-529.

Terishima K., Dobashi M., Maeda K., and Imai Y. (1992). Follicular dendritic cells and iccosomes in germinal center reactions. Sem. Immunol. **4**:267-274.

Tew J.G., and Mandel T.E. (1979). Prolonged antigen half-life in the lymphoid follicles of specifically immunized mice. Immunology **37**:69-76.

Tew J.G., Phipps R.P., and Mandel T.E. (1980). The maintenance and regulation of the humoral response: Persisting antigen and the role of follicular antigen-binding dendritic cells as accessory cells. Immunol. Rev. **53**:175-201.

Tew J.G., Kosco M.H., Burton G.F., and Szakal A.K. (1990). Follicular dendritic cells on accessory cells. Immunol. Rev. **117**:185-211.

Thyphronitis G., Kinoshita T., Inoue K., Schwinle J.E., Tsokos G.C., Metcalf E.S., Finkelman F.D., and Balow J.E. (1991). Modulation of mouse complement receptors 1 & 2 suppress antibody response in vivo. J. Immunol. **147**:224-230.

Tumas-Brundage K., Vora K.A., Giusti A., and Manser T. (1996). Characterization of the *cis*-acting elements required for somatic hypermutation of murine antibody V genes using conventional transgenic and transgene homologous recombination approaches. Sem. Immunol. **8**:141-150.

Von der Heide R.M., and Hunt S.V. (1990). Does the availability of either B cells or CD4$^+$ cells limit germinal center formation? Immunology **69**:487-489.

Vora K.A., and Manser T. (1995). Altering the antibody repertoire via transgene homologous recombination: Evidence for global and clone-autonomous regulation of antigen-driven B-cell differentiation. J. Exp. Med. **181**:271-281.

Vora K.A., Ravetch J.V., and Manser T. (1997). Amplified follicular immune complex deposition in mice lacking the Fc receptor α chain does not alter maturation of the B cell response. J. Immunol. **159:**2116–2124.

Weiss U., and Rajewsky K. (1990). The repertoire of somatic antibody mutants accumulating in the memory compartment after primary immunization is restricted through affinity maturation and mirrors that expressed in the secondary response. J. Exp. Med. **172**:1681-1689.

Xu J., Foy T.M., Lamon J.D., Elliott E.A., Dunn J.J., Waldschmidt T.J., Elsemore J., Noelle R.J., and Flarell R.A. (1994). Mice deficient for CD40 ligand. Immunity **1**:423-431.

Yoshida K., Van Den Berg T.K., and Dijkstra C.D. (1993). Two functionally different follicular dendritic cells in secondary lymphoid follicles of mouse spleen, as revealed by CR1/2 and FcRγII mediated immune complex trapping. Immunology **80**:34-39.

Zheng B., Han S., and Kelsoe G. (1996). T helper cells in murine germinal centers are antigen-specific emigrants that down-regulate Thy-1. J. Exp. Med. **184**:1083-1091.

